# Postmortem Retrograde Contrasted Infusion in Thoracic Duct Outflow: Imaging Effectiveness Analysis

**DOI:** 10.7759/cureus.24224

**Published:** 2022-04-17

**Authors:** Rogério Rodrigo Ramos, Mariane Gabriela Terezani, Elís Claudia Ribeiro Cantarella, Jose Maria Pereira de Godoy, Fernando Batigalia, Luciana Estevam Simonato, Wagner Rafael da Silva, José Martins Pinto Neto, André Wilian Lozano, Nilton Cesar Pezati Boer

**Affiliations:** 1 Department of Health Sciences, University Center of Santa Fé do Sul, Santa Fé do Sul, BRA; 2 Department of Health Sciences, Brasil University, Fernandópolis, BRA; 3 Post-Graduate Program in Imaging Diagnosis and Radiotherapy, São José do Rio Preto School of Medicine (FAMERP), São José do Rio Preto, BRA; 4 Cardiology and Cardiovascular Surgery, São José do Rio Preto School of Medicine (FAMERP), São José do Rio Preto, BRA; 5 Angiology and Vascular Surgery, The Godoy Clinic, São José do Rio Preto, BRA; 6 Human Anatomy, São José do Rio Preto School of Medicine (FAMERP), São José do Rio Preto, BRA

**Keywords:** drainage, dissection, radiology, anatomy, thoracic duct

## Abstract

Background

Thoracic duct (TD) anomaly can be quite variable and dangerous in surgical interventions in the neck region as there are numerous variations in its formation and topography. This highlights the importance of full knowledge about the TD and its anatomical variations. Thus, it is important to emphasize that the lack of anatomical-clinical knowledge or surgical skill during an intervention can significantly hamper successful results. The present study aimed to perform radiopaque contrast infusion into the TD of intact cadavers, either formalinized or refrigerated, to evaluate possible lymphatic architecture patterns via reverse lymphography.

Methodology

TD dissection was performed on 13 cadaveric specimens. After isolating the lymphatic vessel, it was cannulated with an nº 4 urethral probe fixed with cordonnet cotton. Then, a 10 mL syringe was attached to the urethral probe and the radiopaque iodinated contrast was injected into the TD under constant and gradual manual pressure.

Results

TD outflow was detected on the posterior surface of the junction between the internal jugular and the left subclavian veins, either as direct outflow (in 10 cases) or as an arc (in three cases). Reverse contrast progression was impossible in each of the attempts, probably due to valvular resistance and lumen obliteration, which completely prevented pressure infusion into the thoracic and abdominal parts of the TD.

Conclusions

We emphasize the impracticality of obtaining postmortem radiopaque images via retrograde contrast injection into the TD in formalinized or refrigerated bodies.

## Introduction

The thoracic duct (TD) is considered to be a large caliber vessel and is the largest lymphatic vessel in the body, measuring 3 to 5 mm in diameter. Its appearance is similar to the large veins, and it has unidirectional bivalvular valves, which function to prevent thoracic-abdominal lymphatic reflux. It starts at the cisterna chyli, ascends through the aortic hiatus of the diaphragm, follows through the thorax toward the neck, and ends at the junction between the internal jugular vein and the left subclavian vein [1-3].

Histologically, the structure present in TD has a thin elastic fiber layer below the endothelium and a thin smooth muscle cell layer. This layer of smooth muscle is covered by elastic and collagen fibers, which fuse with the surrounding connective tissue, very similar to a relatively underdeveloped adventitia. Its main luminal cells are the lymphocytes, which return to the circulatory system through the TD itself [4,5].

The existing lymph in TD is a mixture of lymphatic fluid originating in the intestine (small and large), liver, lung, and extremities (upper and lower). It has a clear aspect during fasting and a milky aspect after eating. Through it, proteins, hormones, white blood cells, fat molecules, and other nutrients circulate [6,7]. Additionally, the TD collects and carries long-chain fatty acids from the intestine. When lymph transport is impaired, it can lead to obstruction or flawed flow that can result in the chylothorax, chylous ascites, chylopericardium, or chyluria. The most common causes of these conditions are neoplasms, trauma, infection, and venous thrombosis [2,3,8,9].

In addition, conditions associated with malignant tumor cells, especially carcinomas, spread throughout the body through confluent lymphatic vessels to TD. When malignant cells reach a lymph node, they slow down and multiply, and, eventually, metastasize to other regions. Therefore, when surgically removing a cancerous tumor, lymph node examination and removal of both the enlarged lymph nodes in that pathway and the associated lymph vessels are essential for the prevention of secondary tumor growth. If clinical treatment is not available, surgery is indicated [4,10].

TD injury can happen during surgery due to unawareness of its anatomical variations, as well as in extensive procedures such as cervical drainage or trauma, lymph node biopsies, subclavian venous access, and cervical rib resection [11]. TD anomaly can be quite variable as there are numerous variations in its formation and topography [12,13]. Throughout its route, there may also be lymphatic-venous networks that form “plexiform” collateral pathways, which can hinder surgical therapeutic proposals due to the difficulty of intraoperative identification of these structures [14,15].

Anatomical variations in TD outflow can be visualized by conventional lymphangiography or during careful dissection procedures in cadavers [16,17]. Lymphography is an important therapeutic method for lymphatic vessels diagnosis. It consists of a fundamental complimentary examination for defining the best therapeutic strategy due to the variations in TD [18,19].

This denotes the importance of full knowledge about TD and its anatomical variations. Thus, it is important to emphasize that the lack of anatomical-clinical knowledge or surgical skill during an intervention can significantly hamper successful results [20]. The aim of this study was to perform radiopaque contrast infusion into TD of intact cadavers, either formalinized or refrigerated, to evaluate possible lymphatic architecture patterns by reverse lymphography.

## Materials and methods

Three formalinized male cadavers from the Human Anatomy Laboratory of the Universidade Brasil, Campus Fernandópolis, SP, were selected. A second sample was composed of 10 fresh human cadavers (kept in cold storage) from the Death Notification Service (DNS) of São José do Rio Preto (FAMERP - SP) town.

Inclusion criteria included the presence of an intact TD and age equal to or greater than 18 years old. Exclusion criteria were deaths due to cervical, thoracic or abdominal trauma, lymphatic diseases, cadavers weighing more than 100 kg, neoplastic lesion in the cervical, thoracic, or abdominal region, and previous cervical or thoracic surgeries.

To dissect the TD outflow, each body was positioned in dorsal decubitus with a 45º thoracic elevation angle, making a T-incision in the midline near the thyroid cartilage, extended inferiorly to the body of the sternum. Then, through an incision along the clavicle, starting at the sternoclavicular joint up to the acromioclavicular joint on the left side, the skin and subcutaneous tissue were removed. With the clavicle exposed, its periosteum was incised from the left sternoclavicular joint to the acromioclavicular joint to detach the pectoralis major, sternocleidomastoid, sternoioloid, deltoid, subclavian, and trapezius muscles. Then, sternoclavicular disarticulation was performed with lateral clavicular traction for definitive exposure of the deep left subclavian-jugular region.

After careful TD isolation by dissection (with anatomical and curved Kelly® forceps), it was cannulated with an nº 4 urethral tube fixed with cordonnet cotton. After coupling a 10 mL syringe to the urethral tube, 5 mL of Telebrix30® (ioxitalamate meglumine 300 mg/mL) radiopaque iodinated contrast agent was injected into TD under constant and gradual manual pressure. Immediately afterward, under the same technical conditions, 5 mL of the radiopharmaceutical Patent Blue V Guerbet® (25 mg/mL) was infused. For imaging, an Aquilla Plus 300 VMI® Mobile X-ray machine was used [3,21-23].

## Results

**Figure 1:**
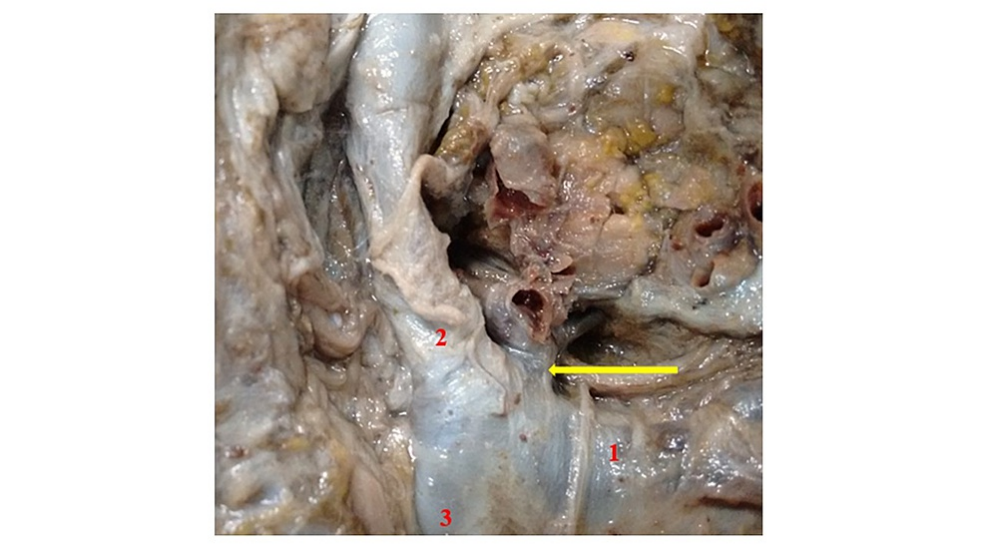
Thoracic duct identification after complete exposure of the deep left jugular-subclavian region in superior view. Notice the direct thoracic duct outflow at the posterior face of the angle between the left subclavian vein and the left internal jugular vein before the formation of the left brachiocephalic vein. (1) Left subclavian vein. (2) Left internal jugular vein. (3) Left brachiocephalic vein. Yellow arrow: thoracic duct.

**Figure 2:**
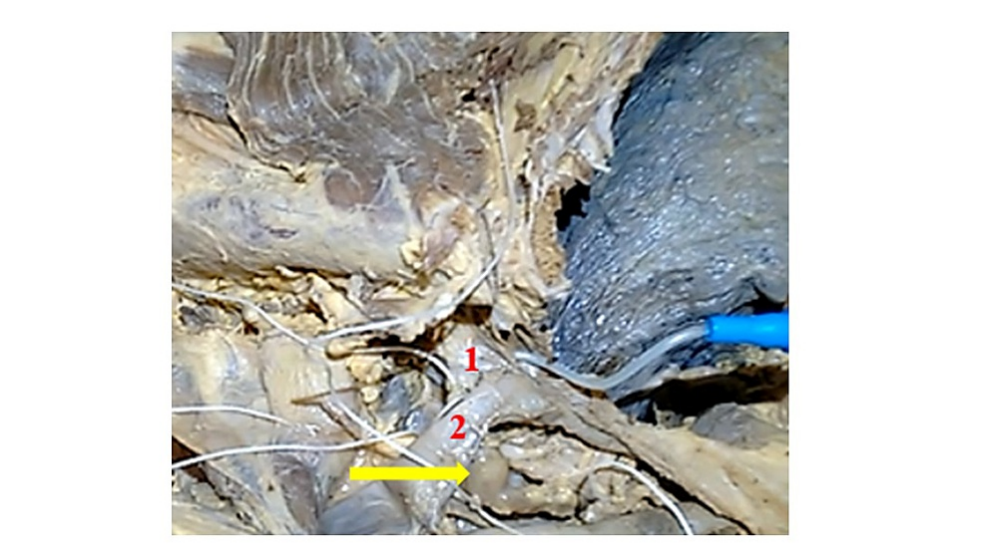
Thoracic duct turgescence deep to its arched outflow at the posterior face of the junction between the left internal jugular and subclavian veins after injection into its lumen of radiopaque contrast (no progression). (1) Left subclavian vein. (2) Left internal jugular. Yellow arrow: thoracic duct turgescence.

In the 13 bodies considered, TD outflow was identified deep in the posterior face of the confluence between the left internal jugular and subclavian veins in 10 cases (Figure 1) or in a posterior arch (in three bodies), in the same topography (Figure 2). After the infusion of both contrasts, in all cases, there was no contrast progression from the TD trunk toward the thoracic cavity, which caused its proximal intumescence (Figure 2). This complication occurred in all cases, despite the slow and constant injection, because the reverse progression of the contrasts was impossible, probably due to valvular resistance and postmortem lumen obliteration. Radiographic series immediately after TD injection did not show contrast agents distribution along the mediastinal lymphatic architecture.

## Discussion

The originality of the present study was to introduce an infusion, in a retrograde manner, of iodate contrast in the TD, to promote visualization of thoracic or abdominal lymphatic vessels and to test the hypothesis of mediastinal or peritoneal lymphatic study from reverse lymphography. However, this study showed proximal swelling by reflux, which prevented contrast progression, even in postmortem conditions, due to the obstructive mechanical action of intraductal lymphatic valves [24].

Another relevant aspect of this study is that drainage of the thoracic duct was not detected directly in the left internal jugular vein or in the left subclavian vein, which confirms the predominance of lymphatic confluence in the jugular-subclavian region in up to 73.3% of the cases [1]. On the other hand, the TD can lead to the left internal jugular vein in 36.2% [25] to 55.5% of cases [26], or even in the left subclavian vein from 17% [27] to 22.3% of the cases [26,28]. Even though TD is the largest lymphatic vessel in the human body with a luminal diameter of up to 5 mm [3], there was no contrast progression within it due to valvular resistance near its outflow into the left venous angle.

Despite the fact that in the present study (of small sample size) no anatomical variations in the TD outflow were detected, in one-third of cases, there may be ductal duplication or even formation of segmental plexuses in one-fifth of the occurrences [29]. A single duct that constitutes TD (as in the present study) is present in up to 21% of cases, and multiple small ducts with individual terminations may still coexist [27]. Such reports may be useful for decisions during diagnostic reasoning or for surgical planning.

## Conclusions

In all cases, the TD outflow was identified on the posterior face of the junction between the internal jugular vein and the left subclavian vein, with direct outflow (in 10 cases) or arched outflow (in three situations). Pressure infusion of radiopaque contrast agents into the TD outflow of formalin-embedded or chilled cadavers was impossible in each attempt, probably due to valvular resistance and lumen obliteration, which contraindicates such a procedure to determine possible lymphatic architecture patterns by postmortem reverse lymphography.
